# Is letrozole during ovarian stimulation useful in breast cancer patients undergoing fertility preservation to reduce early luteal progesterone levels following GnRH-agonist trigger?

**DOI:** 10.1186/s12958-022-00958-7

**Published:** 2022-06-11

**Authors:** Imane Lalami, Julie Labrosse, Isabelle Cedrin-Durnerin, Marjorie Comtet, Claire Vinolas, Fabien Krief, Christophe Sifer, Maeliss Peigne, Michael Grynberg

**Affiliations:** 1 Department of Reproductive Medicine and Fertility Preservation, Hôpital Jean Verdier, Avenue du 14 Juillet, 93140 Bondy, France; 2 Department of Cytogenetic and Reproductive Biology, Hôpital Jean Verdier, Avenue du 14 Juillet, 93140 Bondy, France; 3 University Sorbonne Paris Nord, Paris 13, 93022 Bobigny, France; 4 Department of Reproductive Medicine and Fertility Preservation, Hôpital Antoine Béclère, 157, rue de la Porte de Trivaux, 92140 Clamart, France; 5 University Paris-Sud, Université Paris Saclay, 94276 Le Kremlin Bicêtre, France; 6 Unity Inserm U1133, University Paris-Diderot, 75013 Paris, France

**Keywords:** Ovarian stimulation, Progesterone, Cryopreservation, Estrogen, Luteal phase, Luteinizing hormone

## Abstract

**Background:**

In absence of contraindication, breast cancer patients of reproductive age can undergo fertility preservation with controlled ovarian stimulation for oocyte/embryo cryopreservation before the administration of potentially gonadotoxic treatments. High hormonal levels induced by ovarian stimulation might have an adverse impact on hormone-positive breast cancer. Whether letrozole supplementation during ovarian stimulation (COSTLES) reduces serum progesterone levels after GnRHa trigger remains unknown. We aimed to determine whether COSTLES might be useful for breast cancer patients undergoing fertility preservation to reduce early luteal progesterone levels following GnRH-agonist (GnRHa)trigger.

**Methods:**

All women who underwent COS with GnRH antagonist protocol with GnRHa trigger were included. Serum progesterone level measured 12 h after GnRHa trigger was compared between patients undergoing COS with letrozole supplementation (COSTLES group) and patients undergoing COS without letrozole (Control group) for fertility preservation purposes.

**Results:**

A total of 246 patients were included, of which 84 patients (34.1%) in the COSTLES group and 162 patients (65.6%) in the Control group. All patients in the COSTLES group were BC patients (*n* = 84, 100%), while the Control group included 77 BC patients (47.5%). Patients in the two groups were comparable. The mean number of oocytes recovered and vitrified at metaphase 2 stage did not significantly differ between the two groups. Serum progesterone levels on the day after GnRHa trigger were significantly lower in the COSTLES group (8.6 ± 0.7 *vs.* 10.5 ± 0.5 ng/mL, respectively*, p* < 0.03), as well as serum E2 levels (650.3 ± 57.7 *vs.* 2451.4.0 ± 144.0 pg/mL, respectively, *p* < 0.01). However, the GnRHa-induced LH surge was significantly higher in in the COSTLES group (71.9 ± 4.6 vs. 51.2 ± 2.6 UI/L, respectively, *p* < 0.01).

**Conclusions:**

Our results show that COSTLES for fertility preservation in breast cancer patients using GnRHa trigger reduces serum progesterone levels compared to ovarian stimulation without letrozole. These findings encourage the use of COSTLES in this context to decrease the potential deleterious effect of elevated hormonal levels on hormone-positive breast cancer.

## Background

Breast cancer (BC) is the most frequent cancer in women of reproductive age [1, 2]. Due to substantial advances in treatment and care, the prognosis of young BC patients is constantly improving. The 5-year survival rate of women aged from 15 to 44 years at BC diagnosis reaches 90% [3, 4]. Given better prognoses and the tendency to delay childbearing, it is likely that an increasing number of young BC patients will have a pregnancy desire after BC [5]. Nevertheless, systemic cancer treatments such as chemotherapy may transiently or permanently impair the gonadal function and ovarian reserve of these patients, thus impacting their fertility potential [6–8]. Hence, fertility preservation prior to gonadotoxic treatments has become a key element in the management and care of young cancer patients. Different techniques of fertility preservation can be considered, such as oocyte/embryo cryopreservation after controlled ovarian stimulation (COS), in vitro maturation (IVM) of oocytes, ovarian cortex cryopreservation, or the use of gonadotropin releasing hormone agonist-agonists (GnRHa) during chemotherapy [9, 10].

Oocyte cryopreservation after COS is the first line and most effective method for fertility preservation for postpubertal and adult females prior to cancer treatment in the absence of contraindication [11]. Since COS induces a 10–20-fold increase in oestradiol (E2) levels that might adversely affect hormone-sensitive diseases [12], specific protocols associating aromatase inhibitors such as letrozole and gonadotropins have been developed to maintain serum E2 levels within physiological ranges during COS [13, 14]. However, in addition to E2 levels, high progesterone levels might also have a deleterious effect on BC [15–18]. Some authors raised the hypothesis of a synergistic effect of progesterone and E2 in BC tumorigenesis, in which the receptor activator of nuclear factor kappa-B ligand (RANKL) signalling pathway may be involved [15, 17]. Furthermore, hormonal treatment for menopause combining progesterone and E2 is associated to an increased risk of 26% of BC in women with a history of hysterectomy compared to treatment by E2 only [19].

Altogether, data on progesterone levels during COS with letrozole supplementation (COSTLES) are scarce. The administration of letrozole during COS does not seem to decrease progesterone levels when using antagonist protocols with hCG trigger [20, 21]. However, whether COSTLES has an effect on progesterone levels in case of antagonist protocols with ovulation trigger by GnRHa remains unknown.

The aim of the present study was to determine whether COSTLES reduces early luteal progesterone levels following GnRHa trigger in BC patients undergoing fertility preservation.

## Methods

### Patients

All women undergoing COS for fertility preservation using an antagonist protocol with GnRHa trigger between July 2014 and December 2019 at University Hospital Jean Verdier (France) were included in this retrospective analysis. Patients who had received additional letrozole during the procedure were included in the COSTLES group, while patients who had not received letrozole were included in the Control group. Patients aged below 18 years old or above 42 years old, those who had undergone another type of fertility preservation, cases of metastatic disease and absence of patient consent available were excluded. The study was approved by the institutional review board (CLEA 2021–189) and led according to ethical rules regarding research on patients. For all BC patients, oncologic approval to perform ovarian stimulation for fertility preservation purposes was obtained.

### Ovarian stimulation protocol

In both groups, ovarian stimulation was performed using a GnRH antagonist protocol and administration of recombinant follicle-stimulating hormone (recFSH) (Follitropin alpha; Gonal-F1, Merck-Serono Pharmaceuticals, France). Exogenous FSH therapy was initiated at a dose ranging from 150 to 450 IU/day, S.C, determined according to the age of the patient, body mass index (BMI), serum anti-Müllerian hormone (AMH) levels and antral follicle count (AFC). For BC patients, fertility preservation was initiated either before or after surgery, prior to systemic treatments. Random-start protocols were specifically performed according to the phase of the menstrual cycle. Follicular phase was defined by serum progesterone levels < 1.0 ng/mL and absence of antral follicle > 12 mm in diameter on ultrasound scan. In these situations, recFSH was administered for at least 5 days at initial dosage. From the Day 6 of recFSH therapy onwards, daily recFSH doses were adjusted according to serum E2 levels and/or the number of growing follicles. GnRH antagonist (Ganirelix, Orgalutran1 0.25 mg, S.C, MSD Pharmaceuticals, France) was initiated to prevent a premature LH surge, at Day 6 of gonadotropin stimulation in most cases. Luteal phase was defined by serum progesterone levels > 3.0 ng/mL. For these patients, recFSH was administered in combination with GnRH antagonists for 5 days and further adjusted according to E2 and/or the number of growing follicles.

In the COSTLES group, administration of letrozole (Femara, Novartis Pharma, 5 mg/day orally) was started on the same day as recFSH and stopped on the day of GnRHa trigger. In all patients, GnRHa alone (triptorelin 0.1 mg, Decapeptyl, Ipsen Pharmaceuticals, 0.2 mg, S.C.) was used for final oocyte maturation and ovulation trigger. Ovulation was triggered when at least 3 preovulatory follicles (16–22 mm in diameter, mean of two orthogonal diameters) were observed on ultrasound scan. As previously described by our team, ovulation trigger was performed on follicles 2–3 mm larger in COSTLES cycles compared to non-COSTLES cycles [13, 22]. Vaginal ultrasound-guided oocyte retrieval was performed 36 h after ovulation trigger using local anaesthesia and/or sedation. In accordance with patient desire and medical advice, oocyte and/or embryo (Day 2, Day 3 or Day 5) cryopreservation was performed.

### Ultrasound scans and hormonal measurements

Ovarian reserve and cycle phase were assessed for each patient by ultrasound scan (AFC) and a blood sample measuring serum AMH and progesterone levels. Once COS was started, each patient underwent regular monitoring by ovarian ultrasound scan and blood samples until the day of ovulation trigger by GnRHa. Serum progesterone, LH and E2 levels were measured 12 h after ovulation trigger by GnRHa. Hormonal measurements were performed using commercially available chemo-luminescence immunoassays with an automated Elecsys immunoanalyser (ECLIA, Roche Diagnostics, Meylan, France). The sensitivity of the assay was 5 pg/mL for E2, 0.03 ng/mL for progesterone, and 0.07 IU/L for LH. Intra- and inter-assay coefficients of variation were, respectively, 5 and 10% for E2, 3% and 5% for progesterone, and 2.3% and 2.6% for LH. Ovarian ultrasound scans were performed using a 5.0–9.0 MHz multi-frequency transvaginal probe (Voluson 730 Expert1, General Electric Medical Systems, Paris, France).

### Statistical analysis

The primary endpoint was the progesterone level the morning after GnRHa trigger. Secondary endpoints were: serum LH levels following GnRHa trigger; serum E2 levels following GnRHa trigger; progesterone, LH and E2 levels according to the stimulation start phase (follicular phase/luteal phase); and number of mature oocytes.

Data were expressed in terms of frequencies and percentages or by mean values ± standard deviations (SD). Depending on their distribution, Student or Mann–Whitney tests were used to analyze continuous variables. Statistical analysis was performed using XLSTAT 2019 (Addinsoft, Paris, France) on Microsoft Windows 10. *P*-value < 0.05 was considered statistically significant.

## Results

### Patient and cycle characteristics

**Fig. 1:**
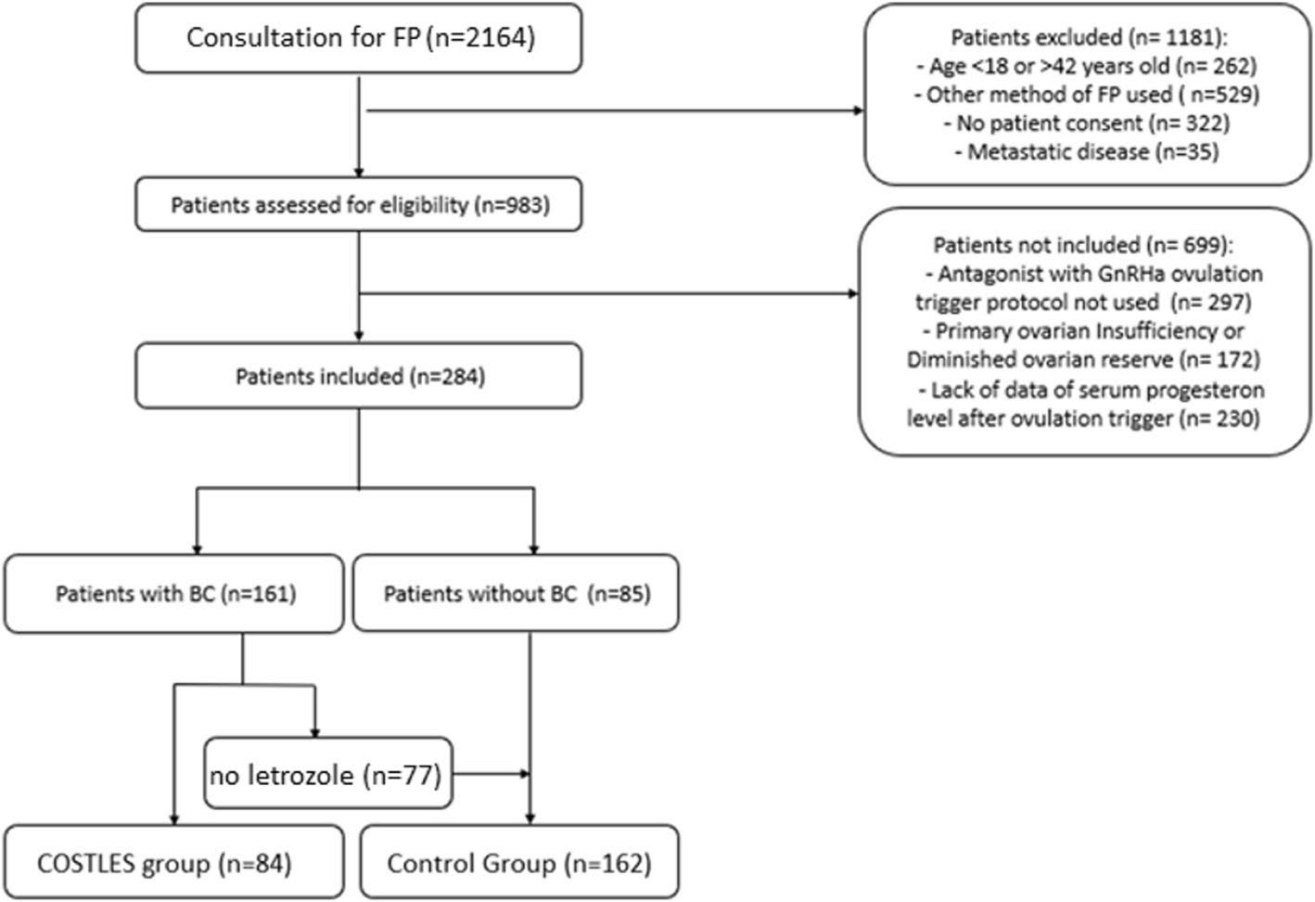
Flow chart. Abbreviations: *FP* fertility preservation, *COSTLES* controlled ovarian stimulation with letrozole supplementation

A total of 246 patients were included in the analysis, of which 84 patients (34.1%) in the COSTLES group and 162 patients (65.6%) in the Control group (Fig. 1). All patients in the COSTLES group were BC patients (n = 84, 100%). In the Control group, 77 patients were BC patients (47.5%). Indications of FP for the other patients of the Control group are detailed Table 1.

**Table 1:** Indications of fertility preservation in the Control group

Indications of fertility preservation	n (%)
Breast cancer patients with no letrozole	77 (47.5)
Premature ovarian insufficiency	45 (27.8)
Endometriosis	25 (15.4)
Auto-immune disease	15 (8.6)

Among BC patients, 122 (75.8%) tumors were positive for estrogen and/or progesterone receptors, while 39 (24.2%) triple negative BC. The majority of BC (97.5%) was classified T1 or T2 according to TNM classification. Distribution was similar between both groups (Table 2).

**Table 2:** Tumor characteristics for breast cancer patients in COSTLES group and control group

Tumor characteristics	COSTLES group *n* = 84	Control group *n* = 77
ER and/or PR positive	63 (75.0%)	59 (76.6%)
T1/T2*	82 (97.6%)	75 (97.4%)
BRCA positive	7 (8.3%)	5 (6.5%)

Abbreviations: *ER* estrogen receptor, *PR* progesterone receptor

^*^according to TNM classification

In the Control group, a majority of patients (*n* = 62/162) had undergone fertility preservation in a cancer context other than BC (blood cell, gastrointestinal, nervous system or cervical cancer, or ovarian borderline tumors), while a minority were cases of fertility preservation for endometriosis (*n* = 9/162) or another benign gynaecologic pathology (*n* = 14/162).

Patients in the COSTLES group and Control group were comparable in terms of age, BMI, AMH levels, AFC and COS characteristics (duration of stimulation, total dose of gonadotropins). The mean number of oocytes recovered (14.2 ± 0.7 *vs.* 14.1 ± 0.9 oocytes, respectively) and vitrified at metaphase 2 stage (10.1 ± 0.6 *vs.* 10.0 ± 0.7 oocytes) did not significantly differ between the two groups (Table 3).

**Table 3:** Patient and cycle characteristics

	COSTLES group	Control group	*p*-value
	*n* = 84	*n* = 162	
Age (years)^a^	31.7 ± 0.4	32.7 ± 0.5	0.17
BMI (kg/m2)^a^	23.6 ± 0.5	23.4 ± 0.4	0.73
Antral follicle count^a^	22.0 ± 1.7	22.2 ± 1.0	0.93
AMH (ng/mL)^a^	2.6 ± 0	2 3.1 ± 0.3	0.18
GnRH antagonist starting day^a^	5.9 ± 0	25 5.8 ± 0.1	0.64
number of days of stimulation^a^	10.4 ± 0	2 10.8 ± 0.2	0.10
Total FSH dose (IU)^a^	2981 ± 237	3168 ± 252	0.30
Number of oocytes recovered^a^	14.2 ± 0.7	14.0 ± 0.8	0.91
Ocytes^a^	10.1 ± 0.6	11.1 ± 0.7	0.28
Number of frozen embryos^a^	7.0 ± 4.3	4.2 ± 2.9	0.20

Abbreviations: *AMH* anti-Müllerian hormone, *BMI* body mass index, *GnRH* gonadotropin releasing hormone, *IU* international unity, *SD* standard deviation

^a^mean ± S

### Outcomes

**Fig. 2:**
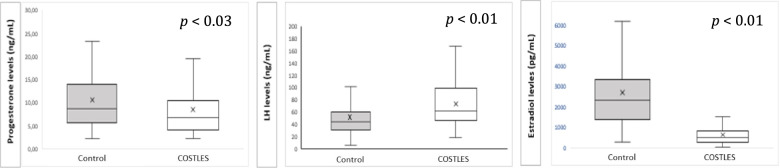
Progesterone, LH and E2 levels measured 12 h after trigger in the COSTLES group and control group. Boxplots represent the median, 25th and 75th percentiles

Serum progesterone levels were significantly lower in the COSTLES group the morning after GnRHa trigger compared to the Control group (8.6 ± 0.7 *vs.* 10.5 ± 0.5 ng/mL, respectively, *p* < 0.03). As expected, the morning after GnRHa trigger, serum E2 levels were also significantly lower in the COSTLES group (650.3 ± 57.7 *vs.* 2451.4.0 ± 144.0 pg/mL, respectively, *p* < 0.01). However, the GnRHa-induced LH surge was significantly higher in the COSTLES group compared to the Control group (71.9 ± 4.6 *vs.* 51.2 ± 2.6 IU/L, respectively, *p* < 0.01) (Fig. 2).

**Fig. 3:**
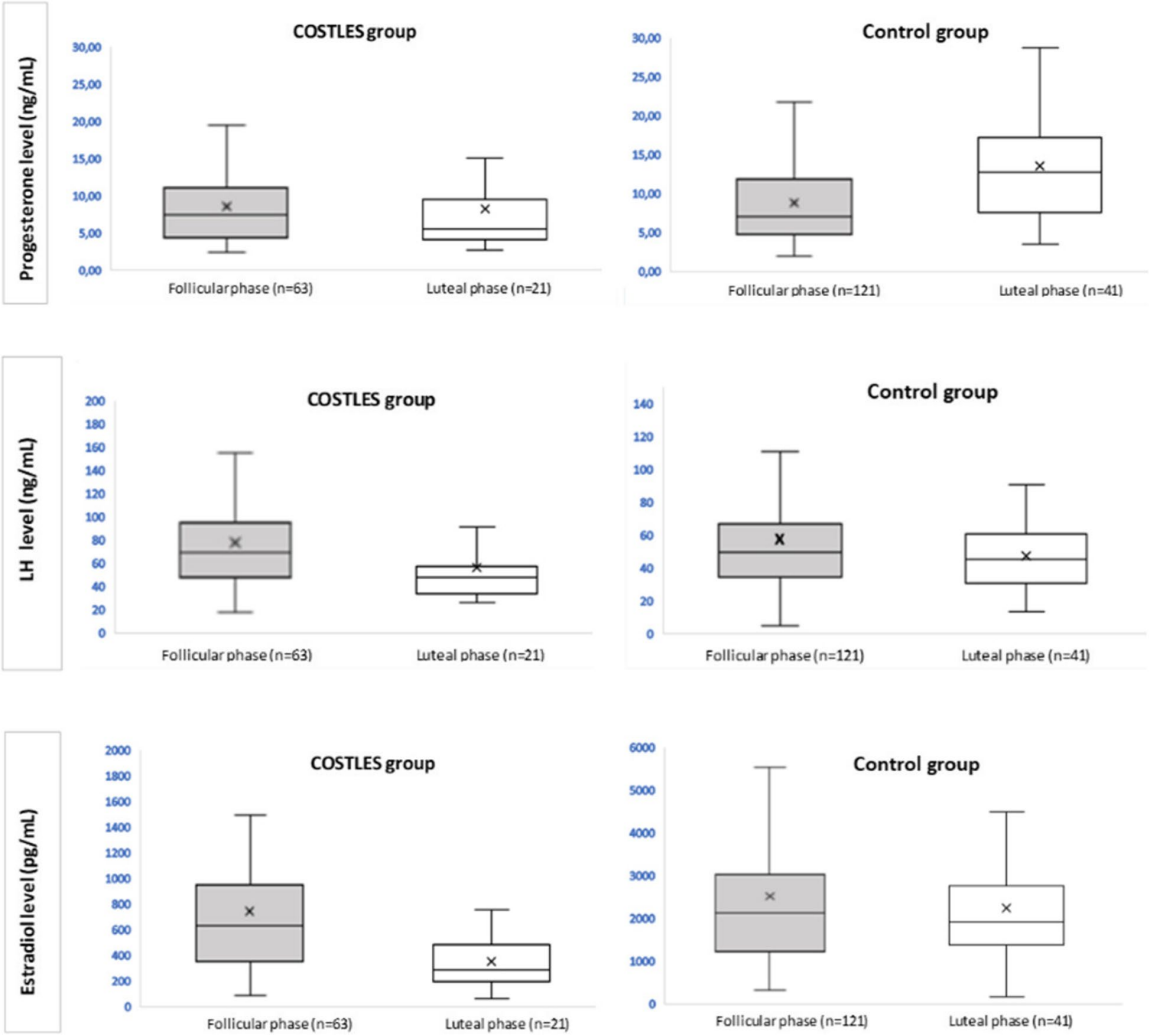
Progesterone, LH and E2 levels according to ovarian stimulation starting phase (follicular or luteal phase) in the COSTLES and control groups

When analyzing hormonal levels according to the stimulation starting phase, serum progesterone levels after GnRHa trigger did not significantly differ in the COSTLES group whether COS had been started in the follicular or luteal phases (8.5 ± 0.8 *vs.* 8.3 ± 1.3, respectively, *p* = 0.9) but was significantly higher when COS had been started in the luteal phase in the Control group (13.6 ± 0.6 *vs.* 9.7 ± 1.3 respectively, *p* = 0.005). Serum E2 levels were significantly higher when COS had been started in the follicular phase in the COSTLES group (752 ± 71.3 *vs.* 351 ± 44.5 pg/mL, *p* = 0.003) and did not differ in the Control group. No significant difference was observed concerning LH levels according to the stimulation starting phase in the COSTLES group nor the Control group (Fig. 3).

## Discussion

Our analysis shows that, in patients undergoing COS with GnRHa trigger in a fertility preservation context, COS with letrozole supplementation (COSTLES) is associated to significantly lower progesterone levels the day after GnRHa trigger compared to COS with no letrozole. E2 levels were also significantly lower in case of letrozole administration compared to without.

To our knowledge, our study is the first to report data on progesterone levels in COSTLES and GnRHa trigger in a large cohort. A previous retrospective observational study reporting 3 cases of fertility preservation patients suggested that progesterone levels were higher after ovarian stimulation with letrozole in the middle luteal phase [21], but in one case, recombinant hCG had been used to trigger ovulation. Moreover, a long GnRHa following trigger (24 to 48 h) had been administered, which may have induced a “flare-up” effect by stimulating progesterone production by the corpus luteum. A prospective observational study of 42 patients assessing progesterone levels on the day of hCG trigger, the day of oocyte retrieval, and 3 and 8 days after oocyte retrieval reported that progesterone levels were similar at all times between letrozole-associated COS and COS without letrozole administration [20]. However, contrarily to our study, all patients had been triggered with recombinant hCG. Altogether, data on progesterone levels in this specific context are scarce and studies so far were performed on small effectives. Furthermore, despite the fact that GnRHa trigger is the most used and recommended in clinical practice in this context, studies so far were based on patients who had received hCG trigger and/or supplementary treatments during or after stimulation which could induce bias.

Lower progesterone levels after GnRHa trigger compared to hCG trigger may be explained by the fact that the use of GnRHa trigger removes the GnRH antagonist from the GnRH receptor and induces a release of endogenous LH and FSH. LH has a shorter half-life than exogenous hCG, thus resulting in a lower luteotropic stimulation with faster luteolysis and lower serum progesterone levels [23, 24]. In COSTLES, lower progesterone levels might be further explained by an increase in progesterone metabolism due to the endogenous inhibition of E2 synthesis. Indeed, letrozole inhibits aromatase that converts androgens into E2, resulting in a decrease in E2 synthesis. In ovarian steroidogenesis, E2 inhibits two enzymes of cytochrome P450 C17 17 α-hydroxylase and 17,20 lyase that metabolizes progesterone to androgens[25]. Hence, lower E2 levels may induce a lower inhibition of these two enzymes, resulting in lower progesterone levels [25]. As such, patients with aromatase deficiency have signs of hyperandogenism [26]. Finally, the higher LH surge in case of letrozole supplementation might stimulate the progesterone metabolism into androgens by its action on cytochrome P450 C17. Indeed, LH could inhibit TGF beta which is a powerful inhibitor of the P450 C17 enzyme that metabolizes progesterone in androgen [27–29].

As expected, E2 levels one day after GnRHa trigger were significantly lower in the COSTLES group. Thus, the higher LH surge following GnRHa administration in the COSTLES group may be associated to a weaker negative feed-back of E2 on the hypothalamic-pituitary axis. The mean number of oocytes recovered and vitrified at metaphase 2 stage were similar in both groups, as related by Oktay et al*.* [13]. In our subgroup analysis of hormone profile according to stimulation starting phase (follicular or luteal), progesterone was significantly higher in case of luteal phase stimulation start in the Control group, with no difference in LH levels. These results are consistent with previous studies observing a correlation between progesterone levels at the end of the follicular phase and levels of progesterone after trigger by GnRHa [30]. E2 levels are similar with results of Pereira et al*.* [31] comparing standard stimulations and random start, in which E2 levels after triggering were similar regardless of the early stage of the stimulation phase (1781 versus 1784 pg/mL). In the COSTLES group, progesterone and LH levels were not significantly different depending on the stimulation start phase. Nevertheless, the E2 levels were significantly different with higher levels when COS was started in the follicular phase.

So far, safety data using COSTLES in BC patients are reassuring. Indeed, a prospective controlled study including 337 BC patients, of which 120 had COSTLES for FP prior to chemotherapy, reported no increased risk of recurrence after a mean follow-up of 5.0 years in case of COSTLES compared to BC patients in which no FP had been performed [32]. Similarly, Azim et al.’s analysis of 215 BC patients, of which 79 had COSTLES, suggested that COSTLES did not adversely affect the risk of reccurrence nor survival outcomes compared to controls after a median follow-up of 23.4 months [33]. Furthermore, the use of a GnRHa trigger in COSTLES cycles appears to have other advantages, as it was shown to significantly reduce the risk of ovarian hyperstimulation syndrome (OHSS) compared to hCG trigger in 129 BC patients undergoing COS for FP purposes (*p* = 0.032) [34]. Consistently, Oktay et al. reported that the incidence rate of OHSS was lower in COSTLES cycles of BC patients triggered by GnRHa compared to hCG [35]. In all, although there is no clinical evidence suggesting that the brief elevation of progesterone levels after hCG trigger impairs the survival of BC patients, it seems that the use of GnRHa trigger should be used in COSTLES cycles given its advantages and the reassuring safety data available so far.

Some limitations should be considered when interpreting results of our study. Progesterone levels were only assessed once, the morning after GnRHa trigger. Hence, our results show that progesterone levels are lower on the day after GnRHa trigger but do not contribute to whether progesterone levels remain consistently lower afterwards. Hence, further studies with several monitoring of progesterone levels would be of interest to confirm our results. Nevertheless, progesterone synthesis is known to increase in the early phase after triggering. Moreover, according to the study by Vuong et al*.* [36], progesterone levels assessed 12 h after recombinant hCG are correlated with progesterone in the mid-luteal phase. Thus, we can assume that progesterone levels in our study might be extrapolated to progesterone levels during the following days in mid-luteal phase. Larger effectives are also required to confirm results of our analysis on hormonal levels depending on the phase during which COS was started.

## Conclusions

Altogether, as progesterone levels may have an adverse impact on hormone-sensitive diseases such as BC, our study suggests that progesterone levels after COS are significantly lower on the day after GnRHa trigger when concomitant letrozole is administered compared to traditional COS with no letrozole. These findings are of particular importance for patients undergoing fertility preservation in this context in order to maximize the chances of fertility preservation success while preventing a potential deleterious effect on the disease. Further studies are warranted to confirm our results and understand their underlying mechanisms.

## Data Availability

All data generated or analysed during this study are included in this published article.

## Abbreviations

BC: Breast cancer
COS: Controlled ovarian stimulation
IVM: In *vitro* maturation
GnRHa: Gonadotropin releasing hormone agonist-agonists
E2: Oestradiol
RANKL: Nuclear factor kappa-B ligand
COSTLES: Controlled ovarian stimulation with letrozole supplementation
RecFSH: Recombinant follicle-stimulating hormone
BMI: Body mass index
AMH: Anti-Müllerian hormone
AFC: Antral follicle count
